# The Effect of Muscular Strength on Depression Symptoms in Adults: A Systematic Review and Meta-Analysis

**DOI:** 10.3390/ijerph17165674

**Published:** 2020-08-06

**Authors:** Adilson Marques, Diego Gomez-Baya, Miguel Peralta, Diana Frasquilho, Teresa Santos, João Martins, Gerson Ferrari, Margarida Gaspar de Matos

**Affiliations:** 1 CIPER, Faculdade de Motricidade Humana, Universidade de Lisboa, 1499-002 Lisbon, Portugal; amarques@fmh.ulisboa.pt (A.M.); jmartins@fmh.ulisboa.pt (J.M.); 2 ISAMB, Faculdade de Medicina, Universidade de Lisboa, 1649-028 Lisbon, Portugal; teresa.santos@universidadeeuropeia.pt (T.S.); mmatos@fmh.ulisboa.pt (M.G.d.M.); 3 Escuela de Doctorado, Universidad de Huelva, 21007 Huelva, Spain; diego.gomez@dpee.uhu.es; 4 Departamento de Psicología Social, Evolutiva y de la Educación, Universidad de Huelva, 21007 Huelva, Spain; 5 Champalimaud Clinical Center, Champalimaud Centre for the Unknown, Champalimaud Foundation, 1400-038 Lisbon, Portugal; diana.frasquilho@research.fchampalimaud.org; 6 Faculdade de Ciências da Saúde e do Desporto, Universidade Europeia, 1500-210 Lisbon, Portugal; 7 Laboratorio de Ciencias de la Actividad Física, el Deporte y la Salud, Facultad de Ciencias Médicas, Universidad de Santiago de Chile, Santiago 8320000, Chile; gerson.demoraes@usach.cl; 8 Faculdade de Motricidade Humana, Universidade de Lisboa, 1499-002 Lisbon, Portugal

**Keywords:** anxiety, mental health, handgrip, fitness

## Abstract

The aim was to systematically review the relationship between muscular strength (MS) and depression symptoms (DS) among adults, and conduct a meta-analysis to determine the pooled odds ratio (OR) for the relationship between MS and DS. The strategies employed in this systematic review followed the Preferred Reporting Items for Systematic Reviews and Meta-Analysis (PRISMA) guidelines. Studies published up to December 2019 were systematically identified by searching in the PubMed, Scopus, and Web of Science electronic databases. Inclusion criteria were: (1) cross-sectional, longitudinal and intervention studies; (2) outcomes included depression or DS; (3) participants were adults and older adults; and (4) the articles were published in English, French, Portuguese, or Spanish. A total of 21 studies were included in the review, totalling 87,508 adults aged ≥18 years, from 26 countries. The systematic review findings suggest that MS has a positive effect on reducing DS. Meta-analysis findings indicate that MS is inversely and significantly related to DS 0.85 (95% CI: 0.80, 0.89). Interventions aiming to improve MS have the potential to promote mental health and prevent depression. Thus, public health professionals could use MS assessment and improvement as a strategy to promote mental health and prevent depression.

## 1. Introduction

Depression is a mental health disorder affecting more than 300 million adults around the world [1]. It is the largest contributor to worldwide disability, and leads to almost one million suicide deaths annually [1]. The most common treatment for depression includes pharmacotherapy and psychotherapy [2,3], which, in the long term, can be expensive and costly for health care systems due to the prevalence of the disorder [4]. In order to address the health problem of depression that affects so many people around the world and to effectively allocate health care spending, it is important to identify additional and effective treatment options.

Regular physical activity increases physical fitness, which is related to a decreased risk of all-cause mortality in healthy people [5]. Physical fitness is a multi-component construct, including cardiorespiratory fitness, muscular fitness, flexibility, balance, and speed. There is longitudinal evidence that cardiorespiratory fitness, a component of physical fitness, is related to depression, preventing it or having a treatment effect [6,7]. Muscular strength is a component of muscular fitness that defines how much strength an individual can voluntarily produce by contracting muscles, and has been associated with a lower risk of mortality in the adult population [8]. Two types of contractions exist: isometric, when the length of the muscle remains the same; and isotonic, when the length of the muscle changes, which can be eccentric or concentric. The most common tests to assess muscular strength are the handgrip strength and the isometric knee extension, which are related to overall strength [9,10]. Both cardiorespiratory fitness and muscular strength are in part genetically determined and in part a result of physical activity and exercise participation. Thus, it is plausible that greater muscular strength also has a positive effect on depression. However, the effect of muscular strength on mental health and depression symptoms is still not clear. Some studies have shown that decreased muscular strength is related to depression [11], while other studies did not find a significant association [12,13].

Muscular strength is positively related to physical function [14]. This is particularly important because a better physical function is significantly associated with less depressive symptoms [15,16]. Moreover, muscular strength is associated with physical activity and exercise habits [17], which are known to be negatively associated with depression symptoms [18,19]. In this line, the possible effects of muscular strength on depression may be mediated by physical activity, which can partly explain this association. Taking into account the significant association between physical activity and the risk of mental illness, it can be hypothesised that better muscular strength may also have a positive effect on reducing or preventing depression. Therefore, a systematic review was conducted, aiming to review the literature regarding the relationship between muscular strength and depression symptoms among adults. Furthermore, a meta-analysis of selected studies was conducted to determine the pooled odds ratio for the relationship between muscular strength and depression symptoms.

## 2. Materials and Methods

The strategies employed in this systematic review followed the Preferred Reporting Items for Systematic Reviews and Meta-Analysis (PRISMA) guidelines [20].

### 2.1. Data Sources and Searches

Studies published up to the 31st of December 2019 were systematically identified by searching in the PubMed, Scopus, and Web of Science electronic databases. The search was performed using the following combination of terms: Handgrip, Hand-Grip or “Hand Strength” or “Grip Strength” or “Muscular Strength” or “Muscular Fitness” or “Muscular Train*” and depression or depressive or “Mental Health” or anxiety or “Psychological Functioning”. Retrieved titles and abstracts were assessed for eligibility for inclusion by two authors. Duplicate entries were removed. Relevant articles were retrieved for a full read. The same two authors reviewed the text of potential studies, and decisions to include or exclude studies in the review were made by consensus.

### 2.2. Study Selection

Articles published up to December 2019 were eligible for inclusion. Specific criteria were: (1) design criterion: cross-sectional, longitudinal and interventional studies; (2) outcome measure criterion: studies were included if the outcomes included depression; (3) participants: adults and older adults; (4) language criterion: articles published in English, French, Portuguese, or Spanish; and (5) exclusion criterion: articles were excluded if they did not meet inclusion criteria or did not include findings related to the inclusion criteria. Published conference proceedings, conference abstracts, and theses or studies including animals were also excluded. Finally, studies focusing on general mental health, anxiety, and mood were excluded because they preclude concluding the isolated effect of muscular strength on depression.

### 2.3. Data Extraction and Quality Assessment

The following information was extracted from each study: author’s name and year of publication, study design, country, sample characteristics (number of participants, sex, age), the instrument for assessing the depression symptoms, the instrument for assessing muscular strength, main results, and study quality. For the intervention studies, the following were extracted: duration of intervention, description of the program, intensity, duration, and frequency. For longitudinal studies, the time of follow-up was also extracted. The methodological quality of the studies was assessed using the Quality Assessment Tool for Quantitative Studies. It is a 19-item checklist, assessing methodological domains: selection bias, study design, confounders, blinding, data-collection methods, withdrawals and dropouts, intervention integrity, and analyses. A global rating was determined based on the scores of each component. Two researchers rated the studies in each domain, as well as the overall quality of each study. Discrepancies were resolved by consensus.

### 2.4. Data Synthesis and Analysis

This review analysed the relationship between muscular strength and depression. The details for each study are presented consistently. Data from all studies were pooled using Review Manager 5.3. For the five clinical trial studies, the instruments used to assess the muscular strength or the depression symptoms were different among the studies. From the 16 observational studies, the data from 9 were used for meta-analysis [11,21,22,23,24,25,26,27,28]. The seven studies not included were eliminated based on the heterogeneity of the association measure between muscular strength and depression symptoms. Two reported the comparison between the effect of high vs. low muscular strength on depression [29,30]; two reported the muscular strength difference between depressed vs. not depressed cases, and the other three reported results stratified by weight status or quartiles of muscular strength [31]. The homogeneity of the odds ratio of the studies included in the meta-analysis was assessed using *I*^2^ statistics. Values of ≥50% were considered substantial heterogeneity [32]. As there was significant heterogeneity, a random effects model was used to calculate the pooled odds ratio. Sensitivity analyses were carried out by successively omitting one study at each turn. A Forest plot was performed to showcase each included study log-odds, odds ratio (95% confidence interval (CI)), standard error and weight as lines, and the pooled odds ratio from all studies as a diamond.

## 3. Results

### 3.1. Literature Search

**Figure 1:**
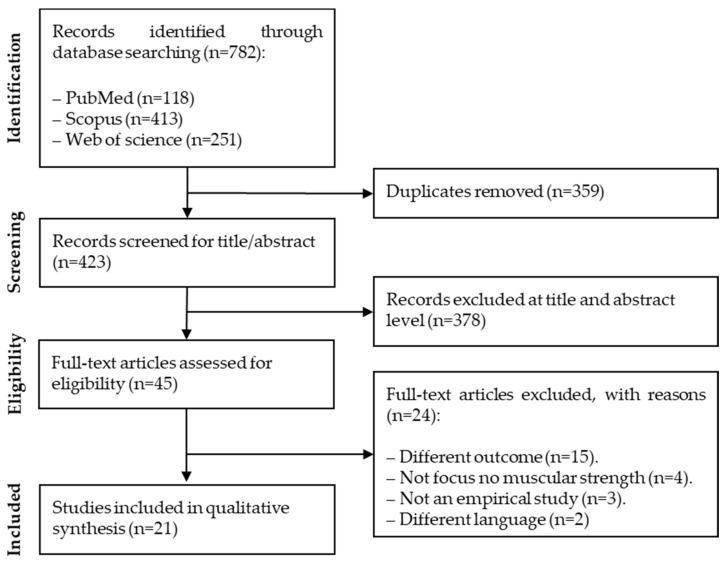
Flow diagram of the study selection.

The primary search in the databases yielded 782 citations. After excluding the duplicates (*n* = 359), the title and abstract of 423 articles were screened. Of these, 378 citations were discarded after reviewing the title and the abstract because it was clear that they did not contain an assessment of muscular strength and depression symptoms. After having read the full text of the remaining 45 citations, 24 were discarded, either because they did not have depression symptoms as the outcome variable (*n* = 15), were not focused on muscular strength (*n* = 4), were not empirical studies (*n* = 3), or were written in a language other than English, French, Portuguese, or Spanish (*n* = 2). The flow diagram is presented in Figure 1.

### 3.2. Study Characteristics

Table 1 presents the studies’ characteristics. A total of 21 studies were included in the review, totalling 87,508 adults aged ≥18 years, from 26 countries. Most studies were cross-sectional (*n* = 12), five were clinical trials, and four were prospective studies. Five studies were performed in South Korea, two in Brazil, two in Spain, two in the United States, and the others were performed in Australia, Belgium, China, Denmark, Finland, Ireland, Japan, Turkey, and the United Kingdom. One study was performed in six countries (China, Ghana, India, Mexico, Russia, South Africa). The Geriatric Depression Scale, Patient Health Questionnaire, and the Beck Depression Inventory were the most used scales to assess the depression symptoms (each scale in four studies). The other scales used were the Center for Epidemiologic Studies Depression Scale, EuroQol Five-Dimension Questionnaire, Hamilton Depression Rating Scale Hopkins’ Symptom Checklist, Mental Health Inventory, and Psychosis Evaluation Tool for Common Use by Caregivers. Seven studies were considered to be of weak methodological quality, seven of moderate quality, and the other seven of strong quality.

### 3.3. Principal Findings

The description of the studies reporting the relationship between muscular strength and depression symptoms is presented in Table 1. The results from two clinical trials [33,34] and one observational study [13] were inconsistent. A relationship between muscular strength and depressive symptoms was not observed. However, in one study, the neurotransmitter factors, such as serotonin, dopamine, epinephrine, and norepinephrine significantly decreased in the strength exercise group but not for the control group [33]. From the other clinical trial studies, the muscular strength decreased the depression symptomatology [35,36,37]. Results demonstrated significant differences in all indicators of depression after completing 12 weeks of training [35], that low muscle strength increased depression symptomatology in patients with fibromyalgia [37], and that strength training intervention significantly decreased depressive symptoms [36]. The other observational studies showed that independently of sex, age, and country, depression symptoms were significantly associated with a reduced handgrip strength [11,21,22,24,27,28,29,30,38,39,40]. Furthermore, adults in the lower tertile or quartile for muscle strength had a significantly higher risk for depressive symptoms compared with those in the third tertile or fourth quartile [23,25,26,31].

### 3.4. Meta-Analysis

**Figure 2:**
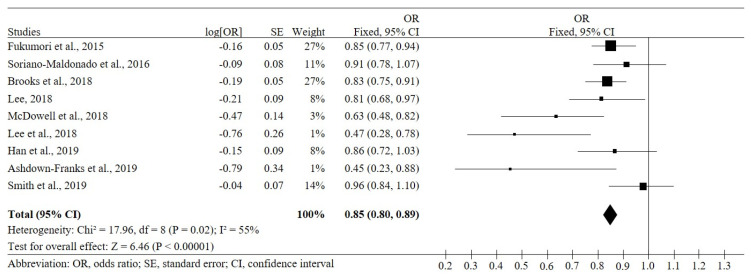
Pooled odds of the association between muscular strength and depression symptoms.

Figure 2 shows the forest plot of the odds ratio and 95% CI of each study and the odds ratio of the random effects model. A moderate heterogeneity among studies was detected (*I*^2^ =55%; χ^2^ = 17.96, *df* = 8; *p* = 0.02). Based on nine observational studies [11,21,22,23,24,25,26,27,28], including 62,831 cases, the presence of depression symptoms was negatively associated with muscular strength. The pooled odds ratio from the random-effects model was 0.85 (95% CI: 0.80, 0.89).

## 4. Discussion

This study sought to review the relationship between muscular strength and depressive symptoms in adults and older adults. Findings suggest that increased muscular fitness may have a beneficial effect on depression symptoms. Furthermore, the meta-analysis findings indicate that muscular fitness is inversely and significantly related to depression symptoms among adults and older adults. This is of importance because depression is the greatest non-communicable disease contributing to the loss of health [1] and it is associated with comorbidity [41], a higher risk of suicide [42], and premature mortality [43].

Muscular strength is recognized as an important health indicator, mainly because it is associated with a lower risk of mortality in the adult and older adults population, regardless of age [8]. Moreover, it is independently associated with cardiometabolic risk and metabolic syndrome in adults and older adults [44,45]. Specifically among older adults, muscular fitness is associated with sarcopenia [46], functional limitations and disabilities [47]. Thus, it is considered a useful marker of frailty in the older adult population [48]. In this systematic review and meta-analysis, muscular fitness was also found to be inversely associated with depressive symptoms. Several mechanisms may explain the observed inverse association between muscular strength and depression symptoms. One possible mechanism is the relationship between muscular strength and sarcopenia [46], functional limitations and disabilities [47]. The relation between muscular strength and these health outcomes may transfer to depression by increased disability, as people with lower muscular strength are less independent and have more difficulties performing activities of daily living [49], which is linked to depression symptoms [50,51]. Moreover, muscular strength is also associated with frailty and health-related quality of life [52]. Another possible explanation is the release of cytokines and myokines into the circulatory systems in response to muscle contraction. It is suggested that myokines might protect against the risk of depression [53]. Furthermore, older adults’ inflammatory profile is associated with frailty and sarcopenia, which can increase the risk of depression [54,55,56].

When examining the association between muscular strength and depression, one must consider the potential mediating role of physical activity. Physical activity, most notably muscle-strengthening activity, is known to be the most important modifiable factor related to muscular strength; therefore, higher muscular strength levels are likely to be related to greater physical activity levels [17]. Furthermore, muscular strength and physical activity share most of the mechanisms through which they may affect depression symptoms. Nevertheless, physical fitness mirrors both participation in physical activity and the state of physiological systems, being suggested that because of that the relationship between physical fitness and health outcomes may be stronger than the relationship between physical activity and those same health outcomes [57]. Moreover, changes in fitness due to participation in physical activity are not immediate and may take weeks to be measurable. Some of the studies included in this review controlled the analysis for physical activity participation and still concluded that muscular strength was associated with depression, independently of physical activity [21,23,24,25,27]. The mediating role of physical activity in the relationship between muscular strength and depression symptoms has not been explored yet, and thus, future research should investigate how much of this relationship is mediated by physical activity.

Depression has one of the greatest disease burdens in several countries worldwide [42]. It is usually treated using pharmacotherapy and psychotherapy [2,3], but both treatment options are expensive and increase the health costs for health care systems [4]. To address this public health problem and possibly reduce the present and future burden of the disease, different strategies are needed. The association between the muscular strength and depression symptoms observed in this review indicates that promoting muscular fitness through physical activity and exercise might be used as a potential strategy to fight depression. Furthermore, a recent systematic review and meta-analysis revealed that resistance training had a moderately positive effect on disability and function in older adults with or at risk of disability [58]. This is important because disability and function are related to depression. Moreover, measures of muscular strength, such as handgrip, could be used as one of several indicators of depression, or for the development of depression symptoms, and its assessment could be promoted in the primary health care systems, as it is already suggested to be for mortality [8]. The early detection and prevention of depression symptoms, and mental health promotion in the health care systems, may lead to healthier populations and reduced health care costs. Moreover, improving muscular fitness would not only promote mental health but also promote cardiometabolic health and the quality of life [45,52,58].

To our knowledge, this is the first systematic review to investigate associations, between muscular fitness and depression symptoms in adults and older adults, using a meta-analysis to determine the pooled odds ratio for the relationship between muscular strength and depression symptoms. Nevertheless, these systematic review and meta-analysis findings should be taken in light of some limitations that must be acknowledged. Most studies used handgrip strength as a measure of muscular strength, while other studies used different measures. This leads to heterogeneity in the muscular strength outcome. Even though the studies were assessed according to their methodological quality, they were not weighed or ranked according to it for the systematic review. Thus, the findings from studies with weaker quality and smaller sample sizes were given no less importance than the findings from studies with strong research designs and larger sample sizes. Furthermore, most included studies were cross-sectional and therefore associations of the temporal direction cannot be certainly assessed. Search terms were selected to identify articles that associate muscular strength and depression. Nonetheless, some articles were eventually left out, because in the title or abstract, they did not have a term that could be associated with muscular strength or depression.

## 5. Conclusions

Muscular strength is inversely and significantly related to depression symptoms among adults and older adults. This may be partially explained by physical activity, but also by the associations between muscular fitness and functional limitations and disabilities, as well as frailty and health-related quality of life, which in turn are related to depression symptoms. Interventions aiming to improve muscular fitness have the potential to promote mental health and prevent depression. Thus, public health professionals could use muscular strength assessment and improvement as a strategy to promote mental health and prevent depression. These findings are of the utmost importance for public policies where the strength of the connection between physical and mental factors, in the promotion of mental health and well-being, is not always duly considered.

## Figures and Tables

**Table 1:** Studies’ characteristics.

Author, Year	Study Design	Country and Sample’s Characteristics	Depression Measure	Muscular Strength Measure	Main Results	Study Quality
Krogh et al., 2009	Clinical trial	Denmark *n* = 165, 122 women, age range = 18–55, mean age 38.9 ± 9.46; strength training (*n* = 55); aerobic training (*n* = 55); relaxation training (*n* = 55).	Hamilton Depression Rating Scale (HAM-D17)	Knee extension; Chest press; Leg press	(Ø) At 4 months, the mean change in the HAM-D17 score was –1.3 (−3.7–1.2; *p* = 0.3) for the strength group vs. the relaxation group. At 12 months, the mean differences in the HAM-D17 score were −0.2 (−2.7–2.3) for the strength group vs. the relaxation group. Findings do not support a biologically mediated effect of strength exercise on symptom severity in depressed patients.	Strong
Suija et al., 2013	Prospective	Finland *n* = 5497, 51% women, age = 31 (all participants were born in 1966).	Hopkins’ Symptom Checklist (HSCL-25)	Trunk extension test; Hand dynamometer	(+) Depressive symptoms were most common among males and females in the lowest quintile group of the trunk extension test and among the males in the lowest quintile group of the handgrip strength compared to the middle quintile group.	Strong
Vancampfort et al., 2013	Cross-sectional	Belgium *n* = 120; schizophrenia: *n* = 80, mean age 36.8 ± 10.0; control: *n* = 40, mean age 37.1 ± 10.3.	Psychosis Evaluation Tool for Common use by Caregivers (PECC)	Standing broad jump; Sit-ups; Handgrip strength	(+) Standing broad jump (−0.35, *p* < 0.01), handgrip strength (−0.28, *p* < 0.01) and sit-ups (−0.33, *p* < 0.01) were inversely associated to depressive symptoms. Low physical fitness was associated with depressive symptoms.	Moderate
Aidar et al., 2014	Clinical trial	Brazil Control group: *n* = 24, 6 men, mean age = 51.7 ± 0.8 Experimental group: *n* = 13, 9 men, mean age = 52.5 ± 7.7.	Beck Depression Inventory (BDI)	Squat; Bench press; Horizontal leg press; Military press; Abdominal crunch; Front lat pull-downs; Lunges	(+) There were significant differences in all indicators of depression between the experimental group and the control group after completing 12 weeks of training. A negative correlation between the strength gains as determined with the one-repetition maximum test and the levels of depression was found.	Weak
Fukumori, 2015	Prospective	Japan *n* = 4314, 58.5% women, age range 40–79, mean age 66.3 ± 9.0.	Mental Health Inventory (MHI-5)	Digital dynamometer (Takei Scientific Instruments Co., Ltd.)	(+) Men and women with lower handgrip strength had higher odds of having depressive symptoms at baseline. Lower handgrip strength was associated with the longitudinal development of depressive symptoms after 1 year (odds ratio (OR) = 1.13, 95%CI = 1.01, 1.27).	Strong
Sener et al., 2016	Clinical trial	Turkey *n* = 79; experimental group: *n* = 39 women with fibromyalgia; mean age 42 ± 10.3 years; control group: *n* = 40 women; mean age 38.3 ± 8.4 years.	Beck Depression Inventory (BDI)	Digital Grip Dynamometer (T.K.K.5401)	(+) In the fibromyalgia group, right and left handgrip strength were moderately negatively correlated with depression scores (r = 0.263 *p* = 0.025; r = 0.233 *p* = 0.048). Low muscle strength increased depression and anxiety symptomatology in patients with fibromyalgia.	Moderate
Soriano-Maldonado et al., 2016	Cross-sectional	Spain *n* = 444 were included in the analysis; mean age 52.0 ± 8.0 years.	Beck Depression Inventory II (BDI II)	Digital Grip Dynamometer (T.K.K. 5101)	(+) The odds of severe symptoms of depression were 4.8% (95% CI: 8.2% to 1.2%; *p* = 0.010) lower for each additional kilogram in the handgrip test among women with fibromyalgia.	Weak
Gopinath et al., 2017	Cross-sectional	Australia *n* = 947 men and women; aged ≥65 years.	Center for Epidemiologic Studies Depression Scale (CES-D-10)	Jamar hand dynamometer (Sammons Preston Inc.)	(Ø) Handgrip strength was not associated with depressive symptoms and quality of life.	Weak
Wu et al., 2017	Cross-sectional	China *n* = 1046, 486 men, 560 women, aged ≥60.	Geriatric Depression Scale (GDS)	Dynamometer (EH101; CAMRY, Guangdong)	(+) Men and women in the lower quartile for muscle strength had a significantly higher risk for depressive symptoms compared with those in the fourth quartile. Muscle strength are inversely associated with depressive symptoms in elderly Chinese.	Weak
Brooks et al., 2018	Cross-sectional	USA *n* = 3421 community-dwelling, non-institutionalized adults; 1660 men, 1761 women; aged ≥60 years, mean age was 69.9 ± 6.9 years.	Patient Health Questionnaire (PHQ-9)	Dynamometer	(+) Depression was significantly associated with reduced handgrip strength in older adults.	Weak
Lee, 2018	Cross-sectional	South Korea *n* = 4810, 2167 men, 2643 women; mean age 50.9 ± 16.7 years.	Depressive mood was assessed using one question: ‘During the past year, did you ever feel so sad or hopeless for 2 weeks or more in a row that you stopped performing usual activities?	Digital Grip Dynamometer (T.K.K.5401)	(+) Handgrip strength was negatively associated with depressive mood (OR = 0.82, 95% CI: 0.69–0.99) and suicidal ideation (OR = 0.73, 95% CI: 0.54–0.99). In a sex-specific relationship, handgrip strength was negatively associated with depressive mood and suicidal ideation among women (OR = 0.71, 95% CI: 0.55–0.93) but not men.	Moderate
Lee et al., 2018	Cross-sectional	South Korea *n* = 4298, 1860 men, 2438 women; age range (19–80 years); subjects were divided into three groups (young adult (19–39 years), middle aged (40–59 years), and elderly (60–80 years).	Patient Health Questionnaire (PHQ-9)	Digital Grip Dynamometer (T.K.K.5401)	(+) Handgrip strength was inversely associated with the PHQ-9 score. The odds ratios of depression symptoms were statistically significant for the participants in the first and second quartile of handgrip strength compared to those with the highest quartile. There was about a 50% mediation effect of EQ5D in the relationship between handgrip strength and depression.	Moderate
McDowell et al., 2018	Prospective	Ireland *n* = 4505, 2544 men, 1961 women; aged ≥50 years.	EuroQol Five-Dimension Questionnaire (EQ5D)	Baseline hydraulic dynamometer	(+) In the total sample, the middle- and high-strength tertiles were significantly associated with 31.5% (*p* = 0.04) and 34.1% (*p* = 0.02) reduced odds of developing depression, respectively. The interaction between sex and strength was not statistically significant.	Strong
Smith et al., 2018	Cross-sectional	USA *n* = 2812, 1380 men, 1432 women, mean age 68.9 ± 0.3 and 69.5 ± 0.3	Center for Epidemiological Studies Depression Scale (CES-D)	Takei Digital Grip Strength Dynamometer	(+) Women with moderate to major depressive symptoms had 1.60 kg (95% CI: 0.91 to 2.30) lower handgrip strength compared to women with minimal or no depressive symptoms. No association was observed in men. Obese men (-3.72 kg, 95% CI:−7.00, −0.43) and women (−1.83 kg, 95% CI:−2.87, −0.78) with moderate to severe depressive symptoms had lower handgrip strength.	Moderate
Ashdown-Franks et al., 2019	Cross-sectional	China, Ghana, India Mexico, Russia, South Africa *n* = 34,129, China 13,175, Ghana 4305, India 6560, Mexico 2313, Russia 3938, South Africa 3838; mean age 62.4 ± 16 years	Patient Health Questionnaire (PHQ-9)	Smedley Handgrip Dynamometer	(+) Individuals with weak handgrip strength had a higher prevalence of depression than those without (8.8% vs. 3.8%; *p* < 0.001). In all countries, weak handgrip strength was associated with a 1.45 (95% CI:1.12–1.88) times higher odds for depression, although some between country differences were noted (particularly in Ghana). Age and sex-stratified analysis showed similar results.	Strong
Han et al., 2019	Cross-sectional	South Korea *n* = 3169, 1451 men, 1718 women; aged 59–69 years, mean age 55 ± 6.25 years.	Patient Health Questionnaire (PHQ-9)	Digital Grip Dynamometer (T.K.K.5401)	(+) Older adults in the lowest tertile of handgrip strength measures were more likely to have experienced depressive symptoms compared to those in the highest tertile.	Moderate
Kim et al., 2019	Clinical trial	South Korea *n* = 21 women, aged 67–81 years, mean age 76.40 ± 3.27 years; control group (*n* = 10; mean age 76.40 ± 3.27 years) and intervention group (*n* = 11, mean age 76.10 ± 3.85 years).	Korean version of the Short form of the Geriatric Depression Scale (SGDS-K)	The exercise program was 3 times/week for 24 weeks. Part I from 1–4 weeks, Part II from 5–8 weeks, Part III from 9–15 weeks, and advanced long-term training from 16 to 24 weeks. The exercise program consists of warm-up (for 10 min), main exercise (for 30–60 min) and warm-down (for 10 min).	(Ø) In neurotransmitter factor, serotonin, dopamine, epinephrine, and norepinephrine were significantly decreased in the strength exercise group but not for the control group. There were no significant differences for both the strength exercise group and control group.	Weak
Laredo-Aguilera et al., 2019	Cross-sectional	Spain *n* = 38 active women, aged >65 years, mean age 72.29 ± 5.21 years.	Spanish version of the Short form of the Geriatric Depression Scale (SGDS-S)	Digital Grip Dynamometer (T.K.K. 5101)	(+) The group with a higher handgrip strength result had a better total score for depression. Significant and negative correlations were found between the handgrip strength and depression.	Weak
Moraes et al., 2019	Clinical trial	Brazil *n* = 27 older adults with major depressive disorder; aerobic training (*n* = 9, 1 man and 8 women, aged 60–78; 70.88 ± 5.94 years), strength training (*n* = 9, 8 men, 1 woman, aged 60–81; 72.89 ± 7.06 years), control group (*n* = 7, 2 men and 5 women, aged 61–77; 69.28 ± 5.28 years).	Hamilton Depression Rating Scale (HAM-D), and Beck Depression Inventory (BDI)	The 1-repetition maximum test was applied. The scale of perception of effort from 6 to 20 was used to quantify the subject’s effort during activity.	(+) The aerobic training and strength training intervention groups showed significant reductions in depressive symptoms from pre to post intervention when compared to the control group.	Moderate
Park et al., 2019	Prospective	South Korea *n* = 13,901, 5996 men, 7905 women; aged > 60 year, mean age 69.54 ± 7.06 years.	Korean version of the Short form of the Geriatric Depression Scale (SGDS-K)	Handgrip Dynamometer (TANITA No. 6103, Tokyo)	(+) Individuals with depression only and individuals with low handgrip strength plus depression had significantly higher risks of all-cause mortality. Men and women with higher handgrip strength had significantly lower depression scores.	Strong
Smith et al., 2019	Cross-sectional	United Kingdom *n* = 3741 community-dwelling, 1257 men, 2484 women; aged 54–89 years, mean age 68.4 years.	Center for Epidemiologic Studies Depression scale (CES-D8)	Hand-held dynamometer	(+) Grip strength was negatively associated with depressive symptoms, when the analysis was adjusted for sex and age. When fully adjusted, the association remained significant but was weaker.	Strong
